# No *Paragonimus *in high-risk groups in Côte d'Ivoire, but considerable prevalence of helminths and intestinal protozoon infections

**DOI:** 10.1186/1756-3305-4-96

**Published:** 2011-06-03

**Authors:** Sylvain G Traoré, Peter Odermatt, Bassirou Bonfoh, Jürg Utzinger, N'da D Aka, Koffi D Adoubryn, Aka Assoumou, Gilles Dreyfuss, Marina Koussémon

**Affiliations:** 1 Laboratoire de Biotechnologie et Microbiologie des Aliments, UFR des Sciences et Technologies des Aliments, Université d'Abobo-Adjamé, 02 BP 801, Abidjan, Côte d'Ivoire; 2 Centre Suisse de Recherches Scientifiques en Côte d'Ivoire, 01 BP 1303, Abidjan, Côte d'Ivoire; 3 Department of Epidemiology and Public Health, Swiss Tropical and Public Health Institute, P.O. Box, CH-4002 Basel, Switzerland; 4 University of Basel, P.O. Box, CH-4003 Basel, Switzerland; 5 Laboratoire de Parasitologie-Mycologie, UFR des Sciences Médicales, Université de Cocody, B.P. V 166, Abidjan, Côte d'Ivoire; 6 UPRES EA n° 3174, Facultés de Médecine et de Pharmacie, F-87025 Limoges, France

## Abstract

**Background:**

Paragonimiasis is a neglected tropical disease caused by an infection with lung flukes that is transmitted through the consumption of undercooked crabs. The disease is often confused with tuberculosis. Paragonimiasis is thought to be endemic in south-western Côte d'Ivoire.

**Methods:**

Two cross-sectional surveys were carried out in the first half of 2009 in patients attending two tuberculosis centres of Abidjan. A third cross-sectional survey was conducted in May 2010 in children of two primary schools in Dabou, where crabs are frequently consumed. Patients with chronic cough provided three sputum samples plus one stool sample. Sputum samples were examined for tuberculosis with an auramine staining technique and for *Paragonimus *eggs using a concentration technique. Stool samples were subjected to the Ritchie technique. Schoolchildren provided a single stool sample, and samples were subjected to the Kato-Katz and an ether-concentration technique. A pre-tested questionnaire was administered to patients and schoolchildren to investigate food consumption habits. Additionally, between June 2009 and August 2010, shellfish were purchased from markets in Abidjan and Dabou and examined for metacercariae.

**Results:**

No human case of paragonimiasis was diagnosed. However, trematode infections were seen in 32 of the 272 shellfish examined (11.8%). Questionnaire results revealed that crab and pig meat is well cooked before consumption. Among the 278 patients with complete data records, 62 had tuberculosis, with a higher prevalence in males than females (28.8% *vs*. 13.9%, χ^2 ^= 8.79, p = 0.003). The prevalence of helminths and intestinal protozoa was 4.6% and 16.9%, respectively. In the school survey, among 166 children with complete data records, the prevalence of helminths and intestinal protozoa was 22.3% and 48.8%, respectively. Boys had significantly higher prevalences of helminths and intestinal protozoa than girls. Hookworm was the predominant helminth species and *Entamoeba coli *was the most common intestinal protozoon species (13.8%).

**Conclusions:**

Not a single case of *Paragonimus *was found in two high-risk groups of Côte d'Ivoire, most likely explained by food consumption habits. However, other helminth and intestinal protozoon infections were common.

## Background

Paragonimiasis belongs to the neglected tropical diseases and is caused by lung flukes of the genus *Paragonimus *[1]. The disease is of considerable public health relevance, particularly in Southeast Asia and South America and in some parts of Africa [2]. It has been estimated that 290 million people are at risk of paragonimiasis and 21 million people are currently infected [3]. In Africa, paragonimiasis has been mainly reported from the West and Central Equatorial region [2,4]. Cameroon and Nigeria are the most affected countries [5]. Paragonimiasis has a complex life cycle with two intermediate hosts; freshwater snails act as the first intermediate host, whereas freshwater crustaceans serve as the second intermediate host, in which the parasites encyst as metacercariae [1,2,6]. Human infection is accomplished by the consumption of raw or insufficiently cooked freshwater crustaceans or meat of paratenic hosts such as pigs or wild boars [1,7]. According to Ripert [8], there are 48 species and subspecies of *Paragonimus*. They have been reported in Asia, the Americas and Africa. Of these, 11 are pathogenic to humans.

The main symptoms of paragonimiasis are chronic cough, with early morning greyish-brown sputum, sometimes with haemoptysis, dyspnoea and chest pain [6,9,10]. Similar symptoms occur due to pulmonary tuberculosis. Hence, the two diseases are often confused [11], which represents a challenge, as the co-existence of paragonimiasis and tuberculosis has been reported [12]. Differential diagnosis is therefore necessary for appropriate patient management. However, in tuberculosis centres of Côte d'Ivoire and elsewhere, differential diagnosis of tuberculosis and paragonimiasis is not pursued.

**Figure 1:**
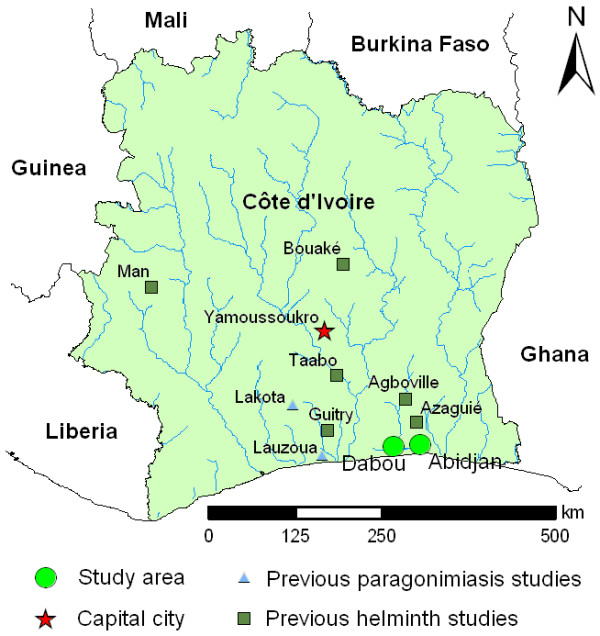
**Map of Côte d'Ivoire, with the locations of the current study in the south of the country (Abidjan and Dabou) and locations where previous studies pertaining to paragonimiasis and other helminth infections have been carried out**.

A first case of paragonimiasis was described in Côte d'Ivoire in 1975 [13]. Subsequently, 14 additional cases have been described until 1999 [14-16]. Epidemiological studies carried out from 2004 to 2006 in the tuberculosis centre of Divo and the regional health centre of Lakota (both located in south-west Côte d'Ivoire) and a rural health centre of Lauzoua Island (in south Côte d'Ivoire) have showed, respectively, no case among 167 patients, 3 cases among 92 patients and 5 cases among 17 patients [5,17,18] (see Figure 1). Nevertheless, paragonimiasis remains a neglected disease in Côte d'Ivoire, both by health professionals and the local population.

There is considerable concern that, in view of the sustained socio-political crisis that Côte d'Ivoire has faced since a coup d'etat in 1999, further exacerbated by an armed conflict that erupted in 2002/2003 [19,20] and a post-election crisis between November 2010 and April 2011, consumption of locally caught crabs of the genus *Callinectes *(second intermediate host of *Paragonimus *spp.) might have increased, and hence altered the risk of paragonimiasis.

The objective of this study was to determine whether paragonimiasis occurs in the south of Côte d'Ivoire, including food consumption habits. We focussed on two high-risk groups, namely patients with a chronic cough of longer than 3 weeks in tuberculosis centres in the economic capital of Côte d'Ivoire, and school-aged children in an area where crab consumption is common. Additionally, shellfish sold on local markets were examined for the presence of metacercariae.

## Methods

### Study area and epidemiological design

Figure 1 shows the locations were the current study was carried out. Two cross-sectional surveys were conducted in March and June 2009 in two tuberculosis centres of Abidjan (Adjamé and Treichville) [21]. A third cross-sectional survey was carried out in two primary schools (N'Gatty and Allaba) in Dabou in May 2010. These two population groups were deliberately selected as they are at high risk of paragonimiasis.

Additionally, cross-sectional surveys were conducted between June 2009 and August 2010 in six markets in Abidjan and the main market in Dabou. Shellfish, including crabs of the genus *Callinectes*, were purchased and transferred to a laboratory for examination of metacercariae and trematode species identification.

### Sample size calculation

In a previous study, a prevalence of 4% of *Paragonimus *spp. was found in three villages in close proximity to the town of Lakota among patients with chronic cough, haemoptysis and/or epilepsy [17]. Allowing for an error of 5%, a precision of 5% regarding the prevalence of infection and a power of 80% revealed a sample size of 57 patients to detect *Paragonimus *spp. We doubled this number and allowed for drop-outs, and hence aimed at screening at least 120 individuals in each of two tuberculosis centres. With regard to the cross-sectional survey in the two primary schools, we also aimed at minimum of 120 children per school. After consulting readily available school lists, we decided to include all 269 schoolchildren attending grades 3 to 6.

Sample size calculation for shellfish was as follows. Based on the study of Aka et al. (2009) [18] in Lauzoua Island, the prevalence of *Callinectes marginatus *crabs found with *Paragonimus *metacercariae was 20%. Allowing for an error of 5%, a precision of 5% regarding infection prevalence and a power of 80%, the estimated sample size was 272 shellfish.

### Ethical considerations

The study was approved by the Ministry of Higher Education and Scientific Research of Côte d'Ivoire (MESRS, decision no. 171), the national tuberculosis control programme and the national ethics committee (CNE decision no. 1176 MSHP). The study was explained to patients who consulted the tuberculosis centres and schoolchildren. We obtained written informed consent from patients aged 16 years and above (verbal consent was given by illiterate people). Parents or guardians of children aged between 5 and 16 years were asked for written informed consent, whereas children assented verbally. Subjects who were deemed too ill for participation and pregnant women were excluded.

The results of the bacteriological tests (tuberculosis centres) and parasitological tests (tuberculosis centres and schools) were communicated to participants by health personnel. Positive tuberculosis patients and those with a *Schistosoma*, soil-transmitted helminth and pathogenic intestinal protozoon infection were referred for treatment at local health clinics. Schoolchildren who where infected with soil-transmitted helminths were treated with a single 400 mg oral dose of albendazole [22].

### Questionnaire survey

After discussing the study protocol with the directors of the two tuberculosis centres, we introduced our study team to their collaborators. The school directors, teachers and parents of participating children were informed about the objectives and procedures of the study. In the tuberculosis centres, we administered a pre-tested questionnaire, consisting of three parts. The first part focused on demographic features (e.g. age, sex and place of residence) and socioeconomic indicators (e.g. type of house and number of household members). Part two pertained to common signs and symptoms, placing particular emphasis on potential sign and symptoms of paragonimiasis (e.g. cough for at least 3 weeks, bloody sputum and chest pain). The third part focussed on risk factors of paragonimiasis (e.g. eating habits, consumption of crabs and pig meat). In addition, questions pertaining to water supply and sanitation were included, as these are important risk factors for helminth and intestinal protozoon infections.

Schoolchildren were interviewed by the teachers. The questionnaire consisted of the same three parts as the one administered to patients seeking care in tuberculosis centres. Additionally, means of crab collection and consumption were investigated.

### Field procedures

In the tuberculosis centre, each patient was assigned a unique identification number (ID) and received three containers for sputum (one sample on day 1, two samples on day 2) and one container for stool collection on day 2. All samples were collected between 08:00 and 11:00 hours and processed the same day in the laboratories of the tuberculosis centres for diagnosis of tuberculosis, and in the parasitology laboratory of the Université de Cocody for determining eggs of *Paragonimus *(and other helminths and intestinal protozoa) in stool and sputum.

In the schools, each child was assigned a unique ID and received one container for collection of a fresh, morning stool sample, collected between 08:00 and 10:00 hours. Stool samples were transferred to the parasitology laboratory of the Centre Suisse de Recherches Scientifiques en Côte d'Ivoire (CSRS) in Abidjan and processed the same day.

Shellfish purchased from six markets in Abidjan (Adjamé, Koumassi, Marcory, Port Bouët, Siporex and Treichville) and the main market of Dabou were transferred to the parasitology laboratory of CSRS and processed the same day.

### Laboratory procedures

At the bacteriology laboratory of the tuberculosis centres, the sputum samples were first subjected to a standard auramine technique [23]. In brief, a small quantity of sputum was spread on a microscope slide, stained with auramine and examined under a fluorescence microscope at high magnification (× 400) by experienced laboratory technicians. The bacilli appear yellow fluorescent against a red background [23]. Sputum samples were then transferred to the parasitology laboratory of the medical school of the Université de Cocody. Sputum samples were examined for the presence of *Paragonimus *eggs. In brief, sputum was diluted with 4% sodium hydroxide (NaOH, one volume of sputum, three volumes of NaOH). Samples were placed in conical tubes for 20 min before being spun in a centrifuge at 1500 *g *for 3 min. The resulting pellets were recovered and examined under a microscope at high magnification (× 400) [17].

At the parasitology laboratory of the medical school of the Université de Cocody, stool samples obtained from patients were subjected to the Ritchie technique [24]. Briefly, approximately 1-2 g of stool was placed in 10 ml of 10% formalin, sieved and then 3 ml of ether was added to the mixture. This stool solution was centrifuged at 1500 *g *for 3 min. The pellet was examined under a microscope at high magnification [24]. Helminths and intestinal protozoa were identified and recorded for each species separately based on morphological and morphometric characteristics of eggs and cysts [25].

At the parasitology laboratory of CSRS, fresh stool samples obtained from schoolchildren were subjected to a single 41.7 mg Kato-Katz thick smear [26,27] and examined for helminths by an experienced laboratory technician. In addition, approximately 1-2 g of stool was fixed in 10 ml of sodium acetate-acetic acid-formalin (SAF) solution. The SAF-fixed stool samples were re-suspended and strained through a medical gauze into a centrifuge tube. The tubes were centrifuged for 1 min at 500 *g*. Seven millilitre of 0.85% NaCl and 3 ml diethyl ether were added to the remaining sediment. Tubes were closed with a rubber stopper, vigorously shaken for 30 sec and centrifuged for 5 min at 2000 *g*. The supernatant was discarded and the resulting sediment examined microscopically for helminths and intestinal protozoa using standard protocols [28].

Shellfish were cut into small pieces with a pair of scissors and ground in a meat grinder before being placed in a jar containing 1 l of tap water. This mixture was manually shaken for 3 min at room temperature and then allowed to stand for 10 min. This procedure was repeated several times until the supernatant became clear. Finally, the sediment was examined under a microscope by an experienced laboratory technician [18].

### Statistical analysis

Data were double entered in SPSS version 10 (IBM Corporation; Somers, NY, USA), and all statistical analyses were done using this software. The prevalence of helminth and intestinal protozoon infections were calculated by comparing the number of positives among those examined. Pearson's χ^2 ^test was used to compare the infection prevalence of tuberculosis by gender. The same test was also used to assess infection prevalence of helminths and intestinal protozoa by gender, both in the tuberculosis centres and the schools.

## Results

### Study compliance

**Figure 2:**
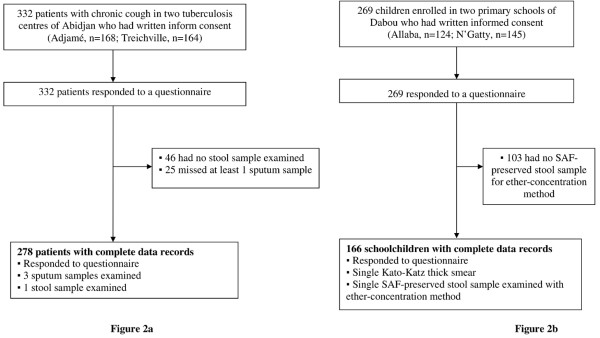
**Flow chart detailing the study participation and adherence of patients in two tuberculosis centres (a) and two schools (b) in south Côte d'Ivoire in 2009 and 2010**. All individuals responded to a questionnaire for risk factors of paragonimiasis and had a stool (and three sputum samples in tuberculosis centres) examined for *Paragonimus *infection and other helminths and intestinal protozoa.

Of 332 patients with chronic cough enrolled in the two tuberculosis centres of Abidjan, 278 (83.7%) provided three sputum samples, one fresh morning stool sample and responded to a questionnaire (Figure 2a). In the two schools surveyed, all of the 269 children invited to participate provided a fresh morning stool and responded to the questionnaire. While all children had a single Kato-Katz thick smear examined, 166 children (61.7%) had sufficiently large stool samples so that 1-2 g could be fixed in SAF for subsequent examination using an ether-concentration method (Figure 2b).

### Questionnaire results

Table 1 summarises the characteristics of the two study populations, placing particular emphasis on risk factors for paragonimiasis. Our questionnaire revealed that from the 278 patients in the two tuberculosis centres, 216 (77.7%) regularly consumed shellfish and 142 (51.1%) regularly consumed pig meat. Most of the respondents boiled crustaceans (94.4%) and two-third cooked pig meat (66.2%) prior to consumption. Three-quarters of the interviewees used tap water as their main source of drinking water. Among the patients in the tuberculosis centres, 248 (89.2%) had chronic cough, 199 (71.6%) had a chest pain and 52 (18.7%) had haemoptysis.

**Table 1:** Characteristics of patients in two tuberculosis centres of Abidjan and schoolchildren in south Côte d'Ivoire who were examined for *Paragonimus *and other helminths and intestinal protozoa in 2009/2010, including risk factors for paragonimiasis and self-reported symptoms.

Characteristics	Patients (n = 278)	Children (n = 166)
**Sex**		
Male	156 (56.1%)	94 (56.6%)
Female	122 (43.9%)	72 (43.4%)

**Age (years)**		
Mean	37.7	11.2
Range	8-80	6-15

**Risk factors of paragonimiasis (and helminths and intestinal protozoa)**		
**Consumption of shellfish**		
**No**	62 (22.3%)	3 (1.8%)
**Yes**	216 (77.7%)	163 (98.2%)
Boiled	204 (94.4%)	163 (100%)
Fried	10 (4.6%)	0
Braised	1 (0.5%)	0
Raw	1 (0.5%)	0

**Consumption of pig meat**		
**No**	136 (48.9%)	7 (4.2%)
**Yes**	142 (51.1%)	159 (95.8%)
Boiled	94 (66.2%)	159 (100%)
Smoked	7 (5.0%)	0
Sausage	2 (1.4%)	0
Fried	1 (0.7%)	0
Boiled and smoked	25 (17.6%)	0
Boiled and braised	4 (2.8%)	0
Boiled and fried	2 (1.4%)	0
Boiled, braised and smoked	7 (5.0%)	0

**Source of drinking water**		
Tap water	208 (74.8%)	96 (57.8%)
Water pump	6 (2.2%)	0
Water supplied by street vendors	57 (20.5%)	1 (0.6%)
Well	6 (2.2%)	69 (41.6%)
Other (creek, river)	1 (0.4%)	0

**Symptoms**		
Chronic cough longer than three weeks	248 (89.2%)	87 (52.4%) *
Chest pain	199 (71.6%)	62 (37.3%)
Haemoptysis	52 (18.7%)	17 (10.2%) **
**Sputum examination**		
BK+	62 (22.3%)	n.d.
BK+ in male	45 (28.8%)	n.d
BK+ in female	17 (13.9%)	n.d

BK+, positive for tuberculosis; n.d., not determined

* children who reported a cough

** children who reported brownish-stained sputum

The school survey revealed that 163 of the children interviewed (98.2%) regularly consumed shellfish and 159 (95.8%) consumed pig meat. Consumption of these two food stuffs was always after boiling. The primary sources of drinking water were taps (57.8%) and wells (41.6%). Eighty-seven among the 166 schoolchildren with complete data records (52.4%) reported a cough, 62 (37.3%) had a chest pain and 17 (10.2%) reported brownish-stained sputum.

### Parasitological and bacteriological results

Sixty-two of the 278 patients with complete data records were diagnosed with pulmonary tuberculosis, owing to an overall prevalence of 22.3%. We found a significant difference in the prevalence of tuberculosis between males and females (28.8% *vs*. 13.9%, χ^2 ^= 8.79, p = 0.003, Table 1). Not a single *Paragonimus *spp. infection was found (Table 2). Overall, 77 (27.7%) patients were infected with at least one helminth or intestinal protozoon species. The prevalence of helminths was 4.6% with species-specific prevalences of *Schistosoma mansoni*, hookworm and *Strongyloides stercoralis *of 1.8%, 1.4% and 1.4%, respectively. The prevalence of intestinal protozoa was 16.9%. *Entamoeba coli *was the predominant species (12.9%), followed by *Endolimax nana, Giardia intestinalis, Iodamoeba bütschlii *and *Chilomastix mesnili *with respective prevalences of 1.8%, 1.4%, 0.3% and 0.3% (Table 2).

**Table 2:** Summary of individuals infected with helminths and intestinal protozoa from two tuberculosis centres in Abidjan and two schools in Dabou, south Côte d'Ivoire, stratified by sex (2009/2010).

	Patients with chronic cough (n = 278)			Schoolchildren (n = 166)		
**Parasite**	**Male**	**Female**	**Total**	**Male**	**Female**	**Total**

**No infection**	116 (74.3%)	85 (69.6%)	201 (72.3%)	54 (57.4%)	54 (75.0%)	108 (65.1%)
**Single infection**	35 (22.4%)	25 (20.5%)	60 (21.6%)	19 (20.2%)	10 (13.9%)	29 (17.5%)
**Helminth**	9 (5.7%)	4 (3.3%)	13 (4.6%)	31 (32.9%)	6 (8.3%)	37 (22.3%)
Hookworm	3 (1.9%)	1 (0.8%)	4 (1.4%)	19 (20.2%)	3 (4.2%)	22 (13.3%)
* Trichuris trichiura*	0	0	0	9 (9.6%)	3 (4.2%)	12 (7.2%)
* Schistosoma mansoni*	2 (1.3%)	3 (2.4%)	5 (1.8%)	0	0	0
*Ascaris lumbricoides*	0	0	0	3 (3.2%)	0	3 (1.8%)
* Strongyloides stercoralis*	4 (2.6%)	0	4 (1.4%)	0	0	0
**Intestinal protozoon**	26 (16.7%)	21 (17.2%)	47 (16.9%)	54 (57.4%)	27 (37.5%)	81 (48.8%)
* Entamoeba coli*	21 (13.4%)	15 (12.3%)	36 (12.9%)	14 (14.9%)	9 (12.5%)	23 (13.8%)
* Endolimax nana*	3 (1.9%)	2 (1.6%)	5 (1.8%)	13 (13.8%)	5 (6.9%)	18 (10.8%)
* Blastocystis hominis*	0	0	0	9 (9.6%)	2 (2.8%)	11 (6.6%)
* Giardia intestinalis*	2(1.3%)	2 (1.6%)	4 (1.4%)	3 (3.2%)	5 (6.9%)	8 (4.8%)
* Entamoeba histolytica/E. dispar*	0	0	0	6 (6.4%)	5 (6.9%)	11 (6.6%)
* Iodamoeba bütschlii*	0	1 (0.8%)	1 (0.3%)	5 (5.3%)	1 (1.4%)	6 (3.6%)
* Chilomastix mesnili*	0	1 (0.8%)	1 (0.3%)	4 (4.3%)	0	4 (2.4%)
**Dual infection**	5 (3.2%)	10 (8.2%)	15 (5.4%)	10 (10.6%)	4 (5.6%)	14 (8.4%)
**Triple infection**	0	2 (1.6%)	2 (0.7%)	4 (4.3%)	2 (2.8%)	6 (3.6%)
**≥ Quadruple infection**	0	0	0	7 (7.4%)	2 (2.8%)	9 (5.4%)

**Total**	156	122	278	94	72	166

In the two schools, no *Paragonimus *spp. infection was diagnosed (Table 2). However, 58 among the 166 children (34.9%) with complete data records (i.e. Kato-Katz thick smear plus SAF-fixed stool sample examined with an ether-concentration technique) were infected with at least one helminth or intestinal protozoon species. The prevalence of helminths was 22.3%. Hookworm was the predominant species (13.3%), followed by *Trichuris trichiura *(7.2%) and *Ascaris lumbricoides *(1.8%). We found a significant difference of helminth infections between boys and girls (32.9% *vs*. 8.3%, χ^2 ^= 14.30, p <0.001).

The prevalence of intestinal protozoa was 48.8% with species-specific prevalences of *E. coli>, E. nana, Blastocystis hominis, Entamoeba histolytica/E. dispar, G. intestinalis, I. bütschlii *and *C. mesnili *of 13.8%, 10.8%, 6.6%, 6.6%, 4.8%, 3.6% and 2.4%, respectively (Table 2). There was a statistically significant difference in the overall prevalence of intestinal protozoon infections between boys and girls (57.4% *vs*. 37.5%, χ^2 ^= 6.49, p = 0.011).

### Results from shellfish examination

Overall, 272 shellfish were examined and this sample consisted of 221 crabs of the genus *Callinectes*, 18 crabs of the genus *Cardiosoma*, 30 shrimps of the genus *Penaeus *and three shrimps of the genus *Macrobrachium*. Metacercariae were found in 32 shellfish, owing to a prevalence of 11.8%. The respective prevalence in crabs of the genus *Callinectes *and *Cardiosoma *were 13.6% and 11.1% (Table 3).

**Table 3:** Infection rate with trematodes (metacercariae) of shellfish purchased from six markets in Abidjan and one market in Dabou in 2009/2010

Genus of shellfish	Number of shellfish analysed	Number (%) of shellfish infected
Crab *Callinectes*	221	30 (13.6)
Crab *Cardisoma*	18	2 (11.1)
Shrimp *Penaeus*	30	0
Shrimp *Macrobrachium*	3	0

**Total**	272	32 (11.8)

## Discussion

In the present study carried out in the southern part of Côte d'Ivoire, West Africa, no *Paragonimus *infections were detected, despite our emphasis to search for this lung fluke among two high-risk groups. Indeed, we screened 278 patients in two tuberculosis centres in the economic capital of Abidjan who suffered from chronic cough. Moreover, we examined 166 children in two schools where consumption of crab meat has been reported. On the other hand, a prevalence of 22.3% of pulmonary tuberculosis was found among patients in the two tuberculosis centres. Helminth infections other than *Paragonimus*, as well as intestinal protozoon infections were common, particularly in schoolchildren. The results on those patients in the tuberculosis centres without pulmonary tuberculosis (n = 216, 77.7%) call for more in-depth studies to elucidate the aetiology of their chronic cough, which requires differential diagnosis.

The absence of paragonimiasis in the two study groups reported here might be explained by culinary practices. While we found trematode infections in crabs purchased from local markets in Abidjan and Dabou, and hence there is a risk of *Paragonimus *infection through consumption of undercooked crab meat, with very few exceptions, study participants reported that they thoroughly boil shellfish and cook pig meat prior to consumption. Indeed, boiling and cooking of shellfish and pig meat destroys metacercariae of *Paragonimus*. Previous research has shown that metacercariae harboured in crabs are destroyed after boiling of shellfish for at least 10 min at a temperature of 55 °C [29].

However one also needs to bear in mind the relatively low sensitivity of the diagnostic techniques employed in the current study [30,31]. To overcome this methodological limitation, we examined three sputum specimens (rather than a single one) from all patients in the tuberculosis centres. None of the sputum specimen was found positive for *Paragonimus *eggs. Nevertheless, paragonimiasis cases might still have been missed, because of the irregular nature of egg shedding by adult parasites, as shown by Miyazaki (1999) [32]. Previous research has shown that *Paragonimus *infections were detected after repeated analysis of sputum and stool samples collected at regular time intervals in the same individual.

With regard to stool examinations, we employed two diagnostic approaches in the school survey: Kato-Katz and an ether-concentration method using SAF-fixed stool samples. Combination of techniques are known to enhance the accuracy of helminth diagnosis [33,34]. Yet, in the present study, no *Paragonimus *infections were found.

Paragonimiasis is difficult to diagnose through stool and sputum examinations using direct diagnostic techniques [12]. Serological techniques such as enzyme-linked immunosorbent assays (ELISA) and immunoblot, produced promising results for paragonimiasis diagnosis [12]. Moreover, the diagnosis is refined with polymerase chain reaction (PCR) using either stool or sputum samples of patients. For example, a study conducted in Cameroon aimed to identify *Paragonimus *species, comparing the sensitivities of serodiagnostic and microscopic methods and evaluated a copro-DNA test for detection of eggs in faeces. Results suggest that serology is considerably more sensitive than sputum examination for the diagnosis of paragonimiasis and a copro-DNA test may be more sensitive than a microscopic search for eggs in faeces [12]. Unfortunately, these diagnostic techniques are not currently available in Côte d'Ivoire and elsewhere in the developing world.

A number of large-scale studies have been carried out in different parts of Côte d'Ivoire, both in schools and entire communities (see Figure 1). Interestingly, in none of these studies were *Paragonimus *eggs detected in stool samples. Although this parasite was not the primary interest in these prior studies, *Paragonimus *eggs are very large, and hence, they would have been detected and noted by the teams of experienced laboratory technicians without any doubt. Parasite eggs (larvae, cyst) of similar and smaller sizes were diagnosed, at times with high prevalence, such as *S. mansoni*, soil-transmitted helminths, *S. stercoralis *and intestinal protozoa [28,35-41].

Although we could not identify cases with paragonimiasis in the current study, we cannot conclude that paragonimiasis is entirely absent from Côte d'Ivoire. Paragonimiasis, similar to other trematode infections, has a highly focal distribution. Typically transmission foci are detected with the diagnosis of a severely sick index case seeking care at local health services [11], which acts as a trigger for more in-depth investigation in the location of origin and a characterisation of the foci [42]. Large-scale surveillance is therefore necessary to detect paragonimiasis foci actively. A simple questionnaire approach has been proven useful for settings in Southeast Asia [43], with which also the case detection rate of tuberculosis patients could be improved [44]. It would be interesting to validate this questionnaire approach in an African setting. Recent studies showed that *Paragonimus *eggs are not destroyed by the widely used Ziehl-Neelsen staining techniques [45]. Therefore, paragonimiasis cases can be detected retrospectively by re-examination of Ziehl-Neelsen stained sputum samples kept at tuberculosis centres. Finally, the surveillance of shellfish, checking for the presence of metacercariae, should be encouraged, which can be integrated in critical point monitoring of food safety activities.

We could not confirm previous reports of *Paragonimus *infection in the south of Côte d'Ivoire. The low endemic level of the infection in animals and humans in this area, the moderate sensitivity of the diagnostic tools utilised and the lack of active surveillance are important limitations. Due to the sustained socio-political crisis, food consumption habits might have changed, altering transmission pathways to humans. Hence, heightened vigilance is required.

## Conclusions

Paragonimiasis is a disease which is found mainly in Southeast Asia. However, cases of paragonimiasis have also been reported from Africa and, given the challenge of diagnosis, it is likely that paragonimiasis is under-reported. Thoroughly boiling shellfish and cooking pig meat, as reported by the participants of the current study, is an effective means for prevention of paragonimiasis. However, some people who use crabs raw or braised and certain target populations (housewives, children and fishermen) who handle or consume them deserve special attention when they seek care at health services due to chronic cough.

## Competing interests

The authors declare that they have no competing interests.

## Authors' contributions

SGT, PO, BB, GD and MK designed the study. SGT, BB, NDA, KDA, AA and MK implemented the study. SGT entered and cleaned the data. SGT, PO and JU analysed and interpreted the data. SGT, PO, BB, JU and MK wrote the paper. All authors read, revised and approved the final version of the manuscript.
